# Effect of the Deposition Time on the Structural, 3D Vertical Growth, and Electrical Conductivity Properties of Electrodeposited Anatase–Rutile Nanostructured Thin Films

**DOI:** 10.3390/mi13081361

**Published:** 2022-08-21

**Authors:** Moisés do Amaral Amâncio, Yonny Romaguera-Barcelay, Robert Saraiva Matos, Marcelo Amanajás Pires, Ariamna María Dip Gandarilla, Marcus Valério Botelho do Nascimento, Francisco Xavier Nobre, Ştefan Ţălu, Henrique Duarte da Fonseca Filho, Walter Ricardo Brito

**Affiliations:** 1 Department of Chemistry, Federal University of Amazonas-UFAM, Manaus 69067-005, AM, Brazil; 2 Department of Physics, Federal University of Amazonas-UFAM, Manaus 69067-005, AM, Brazil; 3 Graduate Program in Materials Science and Engineering, Federal University of Sergipe-UFS, São Cristóvão 49100-000, SE, Brazil; 4 Brazilian Center for Research in Physics—CBPF, Rio de Janeiro 22290-180, RJ, Brazil; 5 Federal Institute of Education, Science and Technology of Amazonas, Coari 69460-000, AM, Brazil; 6 Directorate of Research, Development and Innovation Management (DMCDI), Technical University of Cluj-Napoca, 15 Constantin Daicoviciu St., 400020 Cluj-Napoca, Romania; 7 Laboratory of Nanomaterials Synthesis and Nanoscopy, Department of Physics, Federal University of Amazonas, Manaus 69067-005, AM, Brazil

**Keywords:** electrodeposition, ITO, morphology, thin films, TiO_2_

## Abstract

TiO_2_ time-dependent electrodeposited thin films were synthesized using an electrophoretic apparatus. The XRD analysis revealed that the films could exhibit a crystalline structure composed of ~81% anatase and ~6% rutile after 10 s of deposition, with crystallite size of 15 nm. AFM 3D maps showed that the surfaces obtained between 2 and 10 s of deposition exhibit strong topographical irregularities with long-range and short-range correlations being observed in different surface regions, a trend also observed by the Minkowski functionals. The height-based ISO, as well as specific surface microtexture parameters, showed an overall decrease from 2 to 10 s of deposition, showing a subtle decrease in the vertical growth of the films. The surfaces were also mapped to have low spatial dominant frequencies, which is associated with the similar roughness profile of the films, despite the overall difference in vertical growth observed. The electrical conductivity measurements showed that despite the decrease in topographical roughness, the films acquired a thickness capable of making them increasingly insulating from 2 to 10 s of deposition. Thus, our results prove that the deposition time used during the electrophoretic experiment consistently affects the films’ structure, morphology, and electrical conductivity.

## 1. Introduction

Titanium dioxide (TiO_2_) nanopowders have received much interest due to their use in several technological applications, e.g., in industry and nanotechnology [1]. TiO_2_ is used in many products, and its global demand growth is increasing rapidly along with its low price. It has various advantages, such as high surface tension, specific surface area, magnetic property, lower melting point, good thermal conductivity, and being environmentally friendly [2,3]. TiO_2_ is a polymorphic compound with three mainly crystallographic phases: anatase, rutile, and brookite. They are different in their synthesis and properties, among which rutile and anatase are the most synthesized phases owing to their good thermodynamic characteristics and physical properties. Withal, anatase, and rutile titania exhibit an excellent refractive index, high dielectric constant, higher hiding power, and superior chemical stability [4,5].

TiO_2_ thin films can be deposited using various methods, such as sol–gel dip and spin coating, pulsed laser deposition (PLD), spray pyrolysis, chemical vapor deposition (CVD), and sputtering [6,7]. Among these methods, chemical solution deposition is one of the more promising techniques because it provides higher composition control, lower processing temperatures, shorter fabrication time, and relatively low cost [8]. It is known that the coating deposition method can strongly influence the surface formed and affect properties, which justifies the choice of a method with high composition control.

The comprehension of the growth mechanisms and the study of structure and morphology as a function of the time deposition of the thin films are essential to preparing materials in a measured way for the desired properties. In this regard, studies based on the morphology of the thin films when the time deposition varies help to reveal the growth mechanism of these films [3]. For this purpose, scanning probe microscopy techniques have been employed. Atomic force microscopy (AFM) has been widely used for morphological characterization in real space and for determining thickness, roughness, and particle size in thin films [9]. Moreover, their 3D topographical maps can be used to analyze several other morphological parameters, e.g., spatial, hybrid, feature, functional and volumetric, and geometrical morphology shape and spatial frequencies, which can reveal the unique spatial patterns of the formed topography.

In the present work, we focus on the growth of nanostructured TiO_2_ films on indium tin oxide (ITO) substrates obtained using the electrodeposition technique under different deposition times. This insight aims to analyze the influence of deposition time on the structural, morphological, and electrical conductivity properties of thin films. A correlation between time deposition, structure, surface roughness, growth morphology, and electrical properties of the TiO_2_ films is established, which can help improve the fabrication processes of TiO_2_-based devices. This study aims to obtain optimized conditions for electrodeposited TiO_2_/ITO films at different times to enable photoelectrocatalytic and photovoltaic applications in future studies.

## 2. Materials and Methods

### 2.1. Chemicals and Materials

Potassium hexacyanoferrate (III), potassium chloride, ethyl alcohol (95%), metallic iodine, and titanium dioxide (P25) were purchased from Sigma-Aldrich (San Luis, MO, USA). Potassium hexacyanoferrate (II) trihydrate was obtained from Merck (Darmstadt, HD, Germany). Acetone, acetylacetone (99.5%), ethyl alcohol, and isopropyl alcohol were obtained from Synth (São Paulo, SP, Brazil). ITO/glass substrates (15 Ω/sq) were acquired from Lumtec (Taiwan, China).

### 2.2. Thin Films Electrophoretic Deposition

First, the electrolyte solution was prepared, for which 1.0 g of TiO_2_ and 80 mL of ethyl alcohol were mixed in an Erlenmeyer flask. Then, 100 μL of acetylacetone was added to the system, which remained in magnetic stirring for 24 h. Afterward, 20 mg of metallic iodine was added to the solution, agitated for 30 min, and taken to an ultrasonic bath (Q335D, Quimis, São Paulo, SP, Brazil) for 20 min. The ITO electrodes (2 × 1 cm) were previously cleaned through successive sonication cycles in deionized water, acetone, and isopropyl alcohol for 15 min each stage [9]. Next, specific support for fixing the ITO electrodes was designed, using two connectors, and forming the anode and cathode with a separation of 2 mm. The electrode area to be coated was delimited to 1 cm^2^, and the electrophoretic cell was filled with 10 mL of the electrolytic solution. For the electrodeposition process, the positive and negative poles were connected to the power supply (Agilent E3616A, Santa Clara, CA, USA) and applied a potential of 10 V. The formation of TiO_2_ films occurs by electrostatic attraction of TiO_2_ together with the negative pole electrode (anode), also known as TiO_2_ anodization. Thus, TiO_2_ films were obtained at different times (2, 4, 6, 8, and 10 s) in the TiO_2_ solution. Subsequently, the films were treated thermally at 300 °C for 30 min in a EDG 3P-S muffle from EDG Equipment (São Paulo, SP, Brazil).

### 2.3. Film Characteristics Techniques

#### 2.3.1. X-ray Diffraction Techniques

The X-ray diffraction (XRD) in grazing incident measurements was carried out with a LabX XRD-6000 apparatus from Shimadzu (Japan) and Cu–Kα radiation (λ = 1.5414 Å) source. The measurements were performed from 10 to 60 (2θ) in continuous (0.02/s) and step (0.02/10 s) modes, respectively. The XRD analysis was complemented with the Rietveld refinement technique with the GSAS 1.0 program.

#### 2.3.2. AFM Measurements

An Innova AFM from Bruker (Billerica, MA, USA), operated on a taping mode with a scan rate of 0.5 Hz, was used to make the surface characterization. The samples were scanned in air and 40 ± 1% relative humidity over scanning areas of 5 × 5 μm^2^, with a resolution of 256 × 256 pixels using a silicon cantilever (k = 40 N/m). The feedback control to obtain the best possible images was adapted to each surface and for all the applied scans. The analysis of the images was completed with the WSxM software (Madri, Spain), version 5.0, development 9.1 [10], and, through the images, it is possible to obtain the surface roughness parameters.

#### 2.3.3. Surface Analysis

The complete analysis of the morphology of the films was based on the evaluation of 3D morphological parameters by the International Organization for Standardization (ISO) 25178-2: 2012 standard, whose parameters have their physical meaning largely described in references [11,12,13,14,15]. These parameters were obtained by the MountainsMap^®^ 8.0 (Besançon, France) commercial software from Digital Surf [16]. In summary, we compute and evaluate several parameters, namely, height, feature, spatial, functional, hybrid, volume, and core Sk. Furthermore, we have determined the average power spectrum density (PSD) spectrum of fractal regions of the spectrum using linearized graphs obtained according to the mathematical theory explained by Jacobs et al. [17]. Additionally, the Hurst coefficients of the spectra were calculated using Equation (1), where α is the slope of the linearized curve obtained using the WSxM^©^ 5.0 software [10].

(1) $$Hc=\frac{\alpha -2}{2}$$

Moreover, we know that 3D surface morphology at the micro/nanometric level is characterized by a series of descriptive parameters defined and quantified according to standard ISO 25178-2: 2012, as mentioned above. These descriptive shape parameters do not present a clear quantification of the nanostructured surface model for highlighting fineness characteristics such as arbitrary permutations of topographic positions of different heights. To study the topography more carefully, it was necessary to highlight them by applying integral geometry and morphological descriptors, known as Minkowski functionals (MFs), which characterize both the connectivity (topology) and the content and shape (geometry) of spatial models [18,19,20]. For this purpose, we consider the MFs defined for a convex, compact set *K* ⊂ *R*^3^ via Steiner’s formula. So, let *K* ⊕ *B_r_* be the dilation of the set *K* by a closed ball of radius *r* centered on the origin. The volume *V*^(3)^ (for dimension *d* = 3) of *K* ⊕ *B_r_* can be written as a polynomial function of *r* as follows [19,20]:

(2) $$V^{\left(3\right)}(K⊕B_{r})=\sum_{k=0}^{3}\left(\begin{array}{l} 3\\ k \end{array}\right)W_{k}^{\left(3\right)}(K)r^{k}$$

where *W_k_*^(3)^ is the *k*th Minkowski functional.

The MFs can be expressed using the common descriptors volume *V*, surface area *S*, mean breadth *B*, and Euler–Poincaré characteristic χ by the following relations [18,19]:

(3) $$W_{0}^{\left(3\right)}(K)\;={\;V(K);}^{}{\;W}_{1}^{\left(3\right)}(K)\;=\frac{1}{3}{S(K);\;}^{}W_{2}^{\left(3\right)}(K)\;=\frac{2}{3}\pi \cdot {B(K);}^{}{\;W}_{3}^{\left(3\right)}(K)\;=\frac{4}{3}\pi \cdot \chi \left(K\right)$$

#### 2.3.4. Electrochemical Measurements

The electrochemical measurements were collected in an AUTOLAB PGSTAT 128N (Metrohm Autolab, Utrecht, The Netherlands), controlled with NOVA version 2.1.5 electrochemical analysis software. The electrochemical cell was constituted by three electrodes, using TiO_2_/ITO as the working electrode, Ag/AgCl (sat. KCl) as the reference electrode, and platinum as the auxiliary electrode. The experiments were performed at 22 ± 1 °C, without stirring. The films were characterized by electrochemical impedance spectroscopy (EIS) in a 5 mmol L^−1^ [Fe(CN)6]^4−/3−^ + 0.1 mol L^−1^ KCl solution at 0.2 V, varying the frequency 10 points per decade in the range from 10 kHz to 100 MHz.

## 3. Results and Discussion

**Figure 1:**
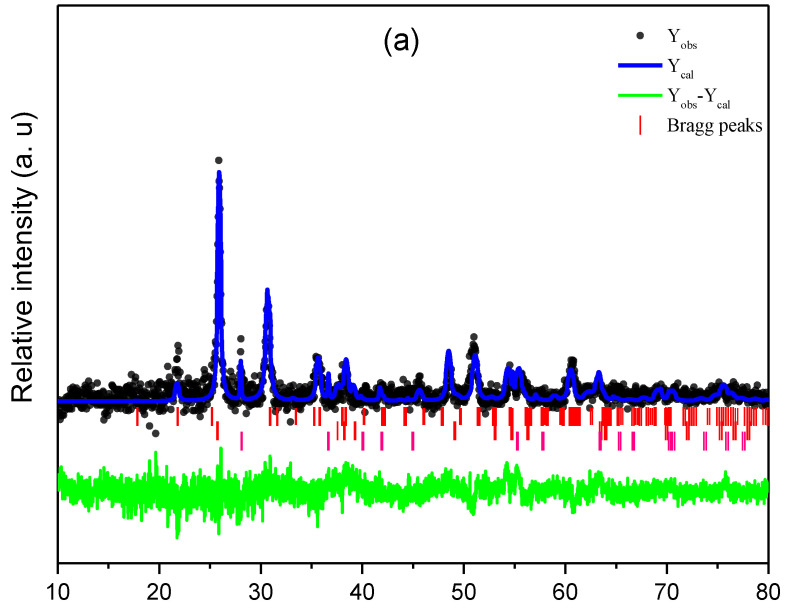

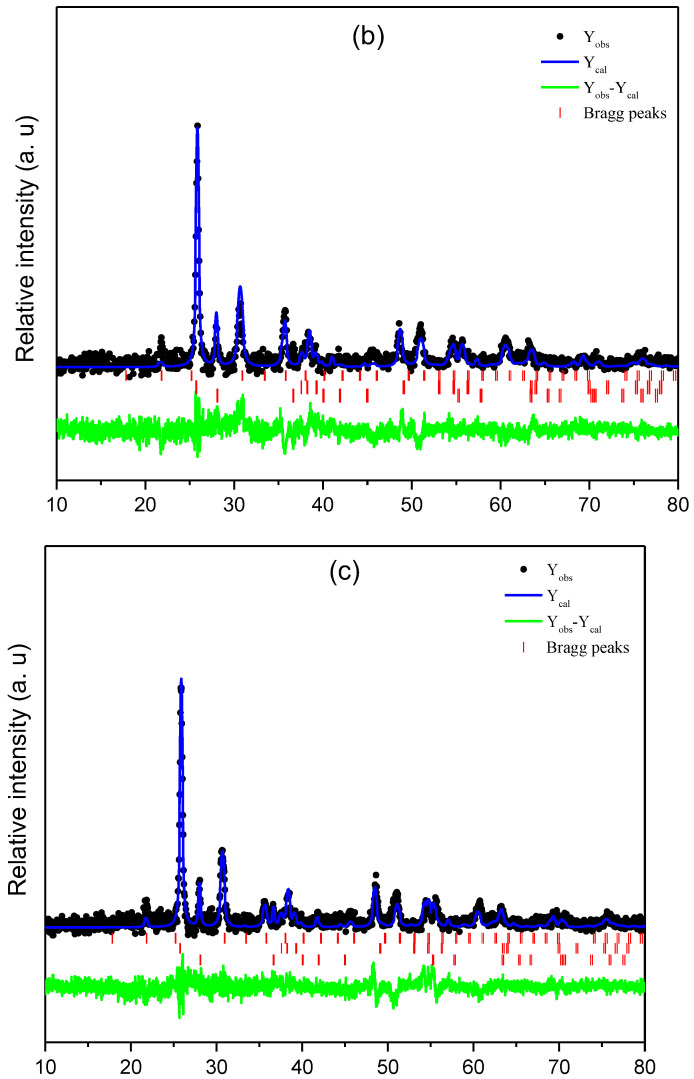

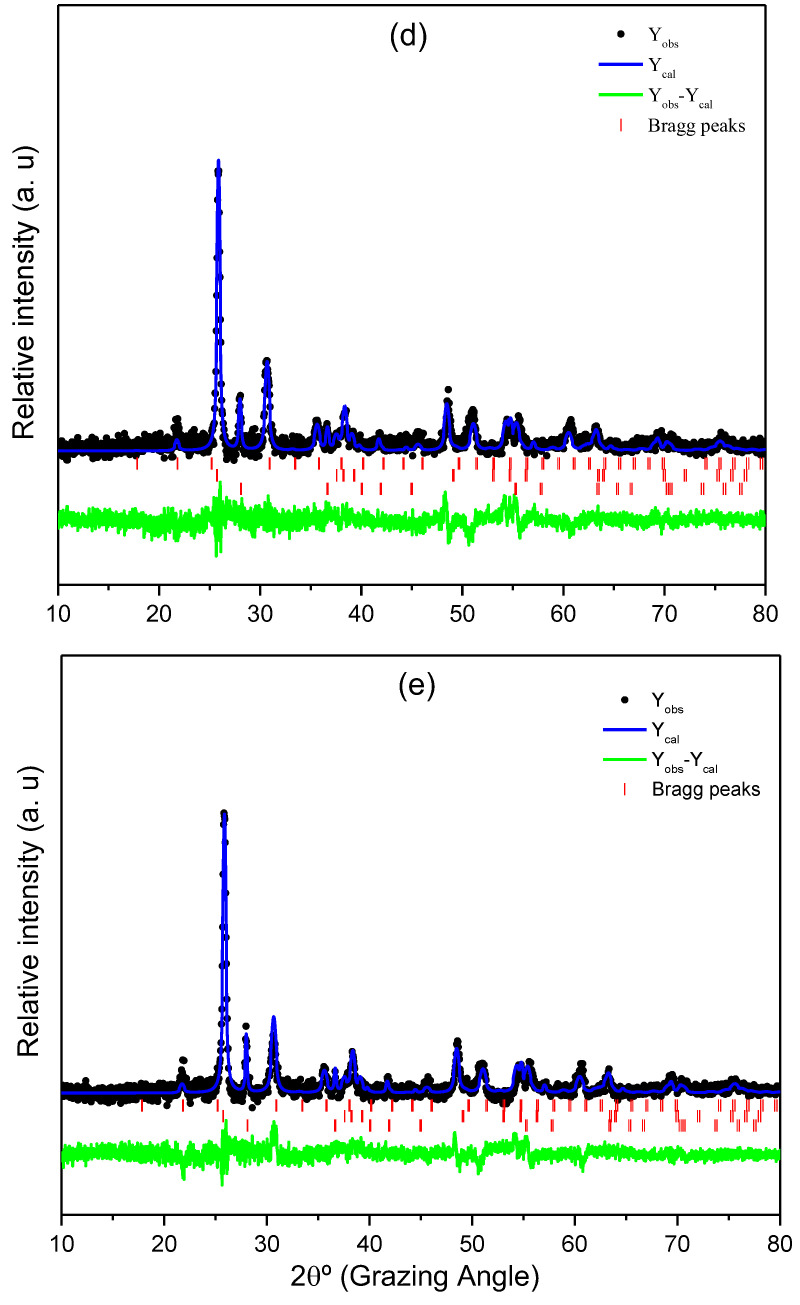
XRD spectra of TiO_2_ thin films at different immersion times: (**a**) 2 s, (**b**) 4 s, (**c**) 6 s, (**d**) 8 s, and (**e**) 10 s. Experimental (Yobs) and theoretical (Yobs) data, in which the residual line (Yobs–Ycal) and Bragg peaks for all samples.

Figure 1 shows the XRD pattern of semiconductor titanium oxide deposited using the immersion method at 300 °C for 2, 4, 6, 8, and 10 s. The diffraction peaks at 25.82°, 38.31°, 41.82°, 48.55°, 55.71°, and 63.34° corresponding to the planes (101), (004), (112), (200), (105), and (215) were observed, indicating the formation of TiO_2_ anatase phase being ~79% of the main phase in all samples [19,20,21]. These signals match well with the peaks previously reported for TiO_2_ in inorganic crystal structure database (ICSD) card No. 9852 and ICSD card No. 24,276 [21,22], which is ~7% of the ITO phase. Other peaks appear in the XRD pattern at 27.97°, 35.72°, and 54.54°, and can be assigned to the planes (110), (101), and (211), revealing the presence of TiO_2_ rutile phase (TiO_2_, ICSD card No. 9161) [23], which is 14% of the other phases. The peaks of indium oxide and tin substrate (indicated as ITO in Figure 1e) were also registered. New phases are not observed in the different immersion times.

XRD spectra of TiO_2_ thin films were refined and used simultaneously for both structures, anatase (ICSD No. 9852) [21,22] and rutile (ICSD No. 9161) [23], assigned to a tetragonal structure, with a space group *I41*/*amd* (141) and space group *P42*/*mnm* (136), respectively. The phase composition analysis revealed that anatase was the major phase at 81% ± 4% in 10 s electrodeposition time, while having lower rutile phase of 6.1%.

Table S1 (Supplementary Information) shows the lattice parameter of TiO_2_ thin films obtained of the refined structure for anatase and rutile in function electrodeposition time. The results verified the correlation between the experimental and theoretical intensities for both samples (Figure 1a,e), as observed in the residual line.

The crystallite size (*D*) for the prepared samples was determined by measuring the broadening of the most intense (best defined) peak of the phase in a diffraction pattern associated with a specific planar reflection within the crystal unit cell according to the Debye–Scherrer equation as follows [24]:

(4) $$D=\frac{k\lambda }{\beta \cdot \cos\theta }$$

where *k* is a correction factor account for particle shapes (in this work, *k* = 9 was used), *λ* is the wavelength of Cu target = 1.5406 Å, *β* is the full width at half maximum (FWHM) of the most intense diffraction peak plane, and *θ* is the Bragg’s angle. The crystalline size calculated for TiO_2_ films with different immersion times evidence that anatase structures have a size of 15 nm for 10 s of immersion compared to rutile structures. Nevertheless, both structures have similar crystalline values in the range from 4 to 6 s, shown in Table S1.

### 3.1. Morphology Analysis

**Figure 2:**
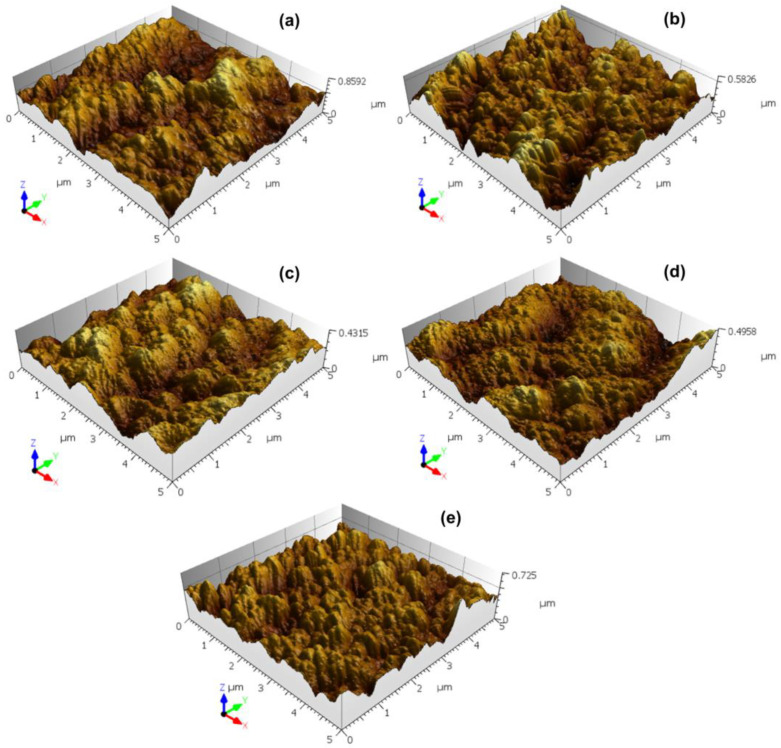
Three-dimensional AFM images of the TiO_2_ thin films with different electrodeposition times: (**a**) 2 s, (**b**) 4 s, (**c**) 6 s, (**d**) 8 s and (**e**) 10 s.

Figure 2 provides the first insights into the topographic properties of the electrodeposited TiO_2_ thin films. The surfaces display an aspect randomly rough on a global scale with some spatial domains, manifesting long-range correlations and other regions exhibiting short-range correlations. Also evident is the hierarchical character of the surface roughness, as several asperities are composed of more asperities that, in turn, are formed with more asperities. Along with the visual information conveyed in Figure 2, we can obtain a quantitative assessment of the topography with the set of height-based parameters, which are provided in Table 1.

Table 1 depicts the temporal values for the root mean square height (Sq), maximum peak height (Sp), maximum pit height (Sv), and maximum height (Sz). In the films obtained with electrodeposition time of 4 to 10 s, an overall consonance between the trends of increase/decrease is noted, related to extreme heights, despite some fluctuations. The concomitant minimum value further emphasizes this at the same intermediate time (t = 6 s).

### 3.2. Advanced Stereometry Evaluation

Table 2 presents the values for the spatial and hybrid parameters [11]. The results did not show significant differences in the autocorrelation length (Sal) for all films, without evidence of considerable increment or decrement in the relative predominance of correlated spatial events. The texture aspect ratio (Str) was another quantity that did not show significant changes over time, suggesting statistical robustness in the magnitude of the surface texture anisotropy from t = 2 s to t = 10 s. Similarly, the texture direction (Std) did not show noticeable changes over time, indicating that the dominant orientation of the surface texture anisotropy persists relatively constant from t = 2 s to t = 10 s.

Now let us focus on the hybrid parameters, which are quantities that have as input the content of the three spatial directions. The values for the root mean square gradient (Sdq) (that is zero for a flat surface) reveal that the samples for t = 6 s present the smallest local steepness. This local property of the surface is corroborated with the interfacial area ratio (Sdr) results, achieving the smallest value for t = 6 s. Such a qualitative agreement is also noted for other time values of Sdq and Sdr, signaling that the local steepness and the relative interfacial surface area incrementally present a coupled response over time in the current set of experiments.

Table 3 conveys information about the temporal behavior of the feature parameters. The density of peaks (Spd) did not show substantial changes over time, suggesting statistical robustness in the number of points (per unit area) for potential contact with other surfaces. On the other hand, the arithmetic means peak curvature (Spc) exhibited strong changes over time where the maximum value occurred for t = 8 s, indicating that the curvature of peaks becomes larger (peaks more rounded) at such an instant. The ten-point height of surface (S10z) and five-point peak height (S5p) attains the minimum value concomitantly at t = 6 s. In contrast, the five-point pit depth (S5v) reaches the minimum value at t = 8 s, suggesting a close temporal behavior between peaks and valleys in the samples. While the mean hill area (Sha) manifested substantial changes, the mean dale area (Sda), mean dale volume (Sdv), and mean hill volume (Shv) presented values without statistical difference. This indicates that the process of formation of the thin films comprised a coupled evolution of the Sda, Sdv, and Shv.

**Figure 3:**
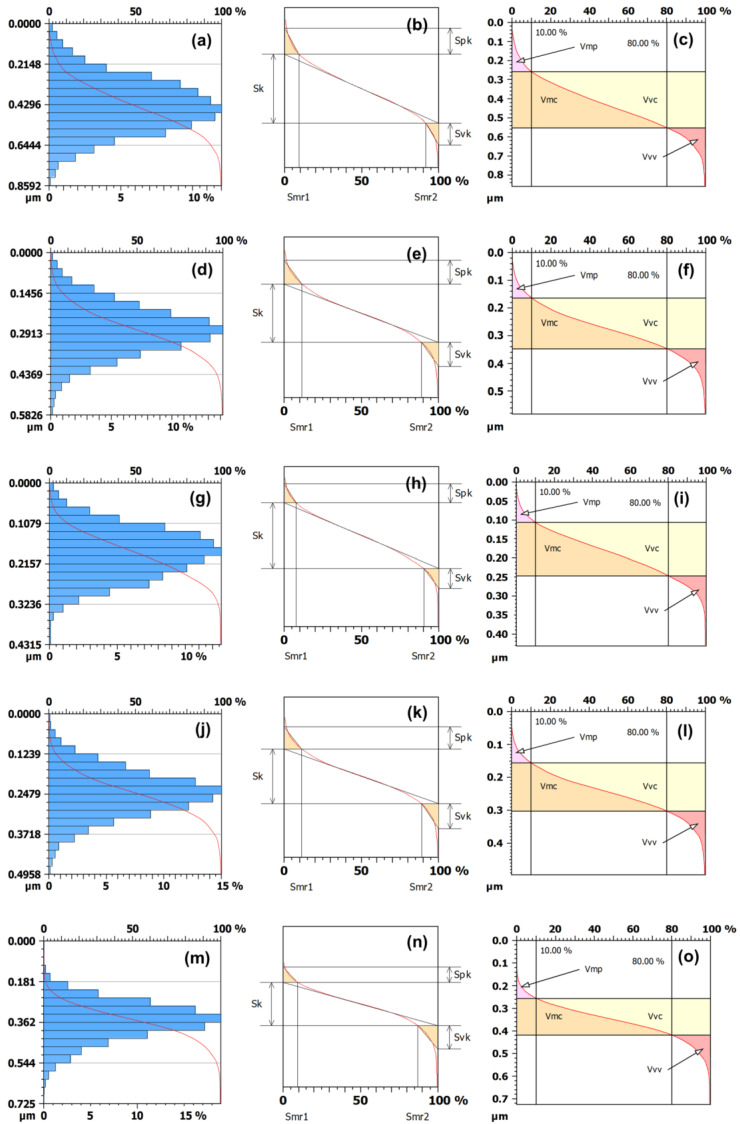
Most relevant Sk and volume parameters representation of (**a**–**c**) 2 s, (**d**–**f**) 4 s, (**g**–**i**) 6 s, (**j**–**m**) 8 s, and (**n**,**o**) 10 s. The horizontal axis in (**c**,**f**) is related to the percentage of occupied volume. The vertical axis is related to depth; 10% and 80% are used to divide the reduced peaks and reduced valleys from the core surface [11].

The panels in Figure 3 reveal that the height histograms have a bell-like shape (with a basis on the *y*-axis). In turn, the red curve overlaid on the histogram is a type of Abbott–Firestone curve [25] that is a suitable statistical method for characterizing surfaces. Such a curve is a cumulative probability density function that, in practice, is estimated from cumulative sums over the height histogram in a way that its minimum value (0%) is obtained for the highest peak, and its maximum is attained for the deepest valley (100%). The results show that the samples of TiO_2_ thin films produce Abbott–Firestone curves with a quite smooth S shape (looking from the *y*-axis). This suggests that for successive depths (from the highest peak), there is a gradual increase in the percentage of the material traversed in relation to the area spanned. The panels {b,c, I–f, h,i, l,m, o,p} provide a statistical interpretation for a plethora of functional parameters [26], which will be discussed further.

Table 4 unveils that the areal material ratio (Smr), peak material portion (Smr1), and valley material portion (Smr2) present variations without statistical difference. This means that the percentage of material that constitutes the peak/valley patterns remains stable over time, despite some fluctuations. It is an obvious general congruence between the patterns of increase/decrease of the inverse areal material ratio (Smc), peak extreme height (Sxp), core roughness depth (Sk), reduced peak height (Spk), and the reduced valley depth (Svk), although there are punctual discrepancies. Similarly, broadly correlated the successive tendencies of growths/declines of dale void volume (Vvv), core void volume (Vvc), peak material volume (Vmp), and core material volume (Vmc). In summary, the results for several functional parameters associated with superficial strata (Smc, Sxp, Sk, Spk, and Svk) and volume (Vvv, Vvc, Vmp, and Vmc) show a correspondence between the temporal variations in distinct portions of the Abbott–Firestone curve obtained from the samples. This points out the overall compatibility between the changes across different slices of the surface morphology.

### 3.3. Power Spectrum Density of the TiO_2_ Thin Films Nanotexture

**Figure 4:**
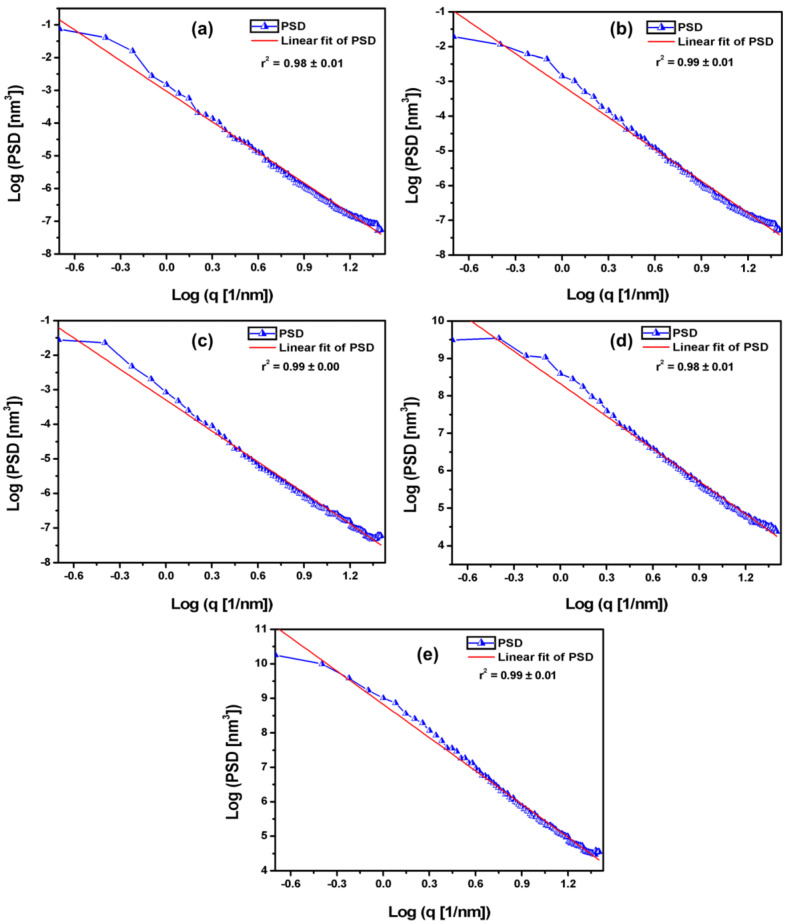
Average power spectrum density of the TiO_2_ thin films with different electrodeposition times: (**a**) 2 s, (**b**) 4 s, (**c**) 6 s, (**d**) 8 s and (**e**) 10 s.

PSD has been an excellent mathematical tool for evaluating patterns on surfaces with different dominant wavelengths [27,28,29]. The linearized graphs of the obtained average PSD spectrum are shown in Figure 4, whose Hurst coefficient values are exposed in Table 5. Although the films exhibited different morphologies, their roughness has similar special frequencies because the spectra are in similar regions. The statistical analysis found no difference between H values, although its mean value has decreased from 2 to 8 s. This suggests that the surface nanotexture does not changed as the deposition time increased, in accordance with Sal, Str, and Std parameters. Due to the film’s uniform deposition, the nanotexture does not showed statistically different patterns of dominant spatial frequency (similar dominant wavelength). This nanotexture characteristic can also favor some similar physical properties, in addition to their homogeneity, as observed by Barcelay et al. [15].

### 3.4. Minkowski Functionals

**Figure 5:**
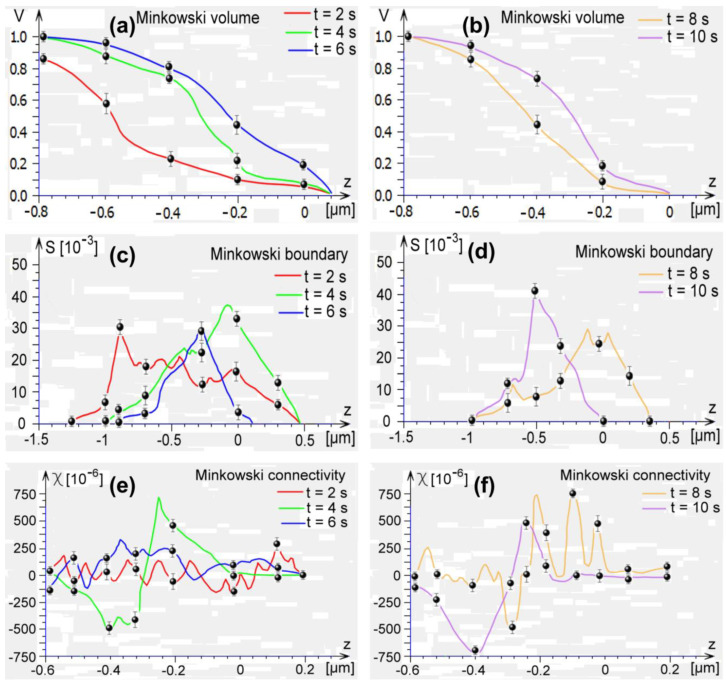
Minkowski volume of the thin films for: (**a**) 2 s, 4 s, and 6 s; (**b**) 8 s, and 10 s; Minkowski boundary of the thin films for: (**c**) 2 s, 4 s, and 6 s; (**d**) 8 s, and 10 s; Minkowski connectivity of the thin films for: (**e**) 2 s, 4 s, and 6 s; (**f**) 8 s, and 10 s.

The MFs of AFM images were calculated and averaged over various positions on each surface. The resulting graphs for Minkowski volume *V*, Minkowski boundary *S*, and Minkowski connectivity χ are plotted in Figure 5. As can be seen, three descriptors (Minkowski volume, Minkowski limit, and Minkowski connectivity) show a different graphical evolution, which highlights a distinct 3D morphology of the surface texture with different types of nanopatterned templates. For the Minkowski volume *V* (Figure 5a,b), the lowest values are for samples with t = 2 s, and the highest values correspond to samples with t = 10 s. The Minkowski boundary *S* (Figure 5c,d) presents a maximum peak in the samples with t = 10 s. Furthermore, the Minkowski connectivity χ (Figure 5e,f) describes the topological structure of the surfaces’ patterns. All graphs have an oscillated form with values dispersed in a wide range of heights. There is a maximum peak for samples with t = 8 s, while the minimum value is associated with samples with t = 10 s.

### 3.5. Electrochemical Impedance Spectroscopy of the TiO_2_ Films

**Figure 6:**
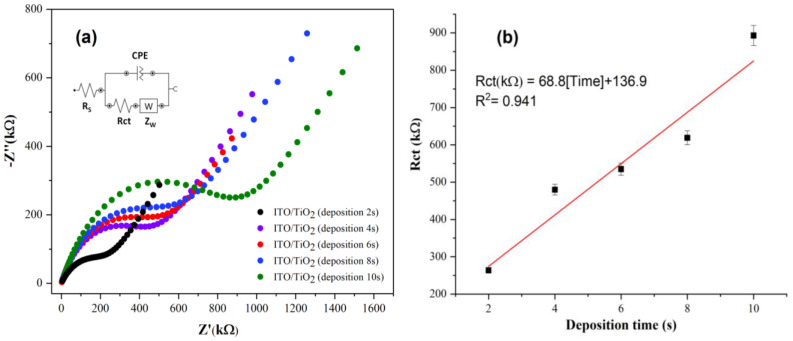
Electrochemical impedance spectroscopy in [Fe(CN)_6_]^4−/3−^ + KCl solution. (**a**) Nyquist diagrams of ITO/TiO_2_ with the application of different deposition times, and (**b**) calibration curve (Plots of Rct versus deposition time).

The results of EIS are represented as Nyquist graphs (Figure 6), which include a semicircle in high-frequency ranges representing the charge transfer resistance (Rct) and a linear part at lower frequencies representing the diffusion process. The resistance values (Rct) were calculated using the Randles equivalent circuit (inset Figure 6), where Rs is the solution and connectors resistance, Zw is the Warburg impedance that corresponds to mass transfer of the redox species to the electrode, and constant phase element (CPE) is the impedance of the electrode/solution interface and is associated with the formation of the double electrical layer [30].

As can be observed (Figure 6a), the semicircle at high frequencies increased with the deposition time due to the formation of a thicker TiO_2_ layer, attributing insulating properties to the film. The calibration curve was obtained by plotting resistance (Rct) (kΩ) versus deposition time (Figure 6b), where a linear behavior is observed. The equation of regression was defined as Rct (kΩ) = 68.8 [deposition time] + 136.9, with R^2^ = 0.941.

## 4. Conclusions

In summary, anatase–rutile time-dependent electrodeposited thin films were successfully obtained in this work. The films present a tetragonal crystalline structure ascribed to the anatase phase (81%), but also exhibit small amounts (~6%) of rutile after 10 s of deposition. The crystallite size of all films was found to be around 15 nm. The long- and short-range correlations found along the film’s surface are assigned to the main phenomena that characterize the strong topographical irregularities of the films. Despite this, the vertical growth decreases as the deposition time increases, showing that the surface of the films becomes smoother. This trend was confirmed by the analysis of Minkowski functionals. PSD results indicate that all films are dominated by dominant low spatial frequencies, which can be attributed to similar surface microtexture, although the surfaces have different morphologies. The electrical conductivity tests showed that the films become more insulating as the deposition time increases, which notably occurs thanks to the increase in the thickness of the oxide layer that is produced on the ITO substrate. Our findings prove that the structure, morphology, and electrical conductivity of anatase–rutile films could be controlled by the deposition time employed in the electrophoretic essay, showing that this physical parameter plays an important role in the film’s physical properties.

## Figures and Tables

**Table 1:** Surface parameters of the thin films, according to ISO 25178-2:2012.

Par [μm]	Electrodeposition Time				
	2 s	4 s	6 s	8 s	10 s
Sq	0.122 ± 0.019	0.108 ± 0.023	0.068 ± 0.010	0.068 ± 0.006	0.091 ± 0.008
Sp	0.411 ± 0.049	0.358 ± 0.085	0.233 ± 0.058	0.267 ± 0.023	0.336 ± 0.076
Sv	0.403 ± 0.046	0.471 ± 0.148	0.234 ± 0.020	0.239 ± 0.014	0.353 ± 0.067
Sz	0.814 ± 0.088	0.829 ± 0.233	0.467 ± 0.070	0.506 ± 0.031	0.688 ± 0.137

**Table 2:** Spatial and hybrid parameters of the thin films, in accordance with ISO 25178-2:2012.

Par	Unit	Electrodeposition Time				
		2 s	4 s	6 s	8 s	10 s
***Spatial***						
Sal *	[μm]	0.432 ± 0.044	0.353 ± 0.037	0.383 ± 0.021	0.375 ± 0.050	0.338 ± 0.049
Str *	[-]	0.472 ± 0.011	0.427 ± 0.136	0.394 ± 0.191	0.603 ± 0.069	0.613 ± 0.126
Std *	[*°*]	123.94 ± 67.30	51.75 ± 72.72	52.00 ± 69.28	99.87 ± 77.46	118.44 ± 63.85
***Hybrid***						
Sdq	[-]	961.95 ± 54.25	978.06 ± 114.74	495.91 ± 31.63	672.96 ± 141.40	878.85 ± 80.38
Sdr	[%]	75.33 ± 3.09	75.75 ± 6.29	42.00 ± 2.37	55.17 ± 11.35	70.08 ± 5.40

* Samples without significant difference, one-way ANOVA and Tukey’s test (*p* < 0.05).

**Table 3:** Feature parameters of the thin films, in accordance with ISO 25178-2:2012.

Par	Unit	Electrodeposition Time				
		2 s	4 s	6 s	8 s	10 s
Spd *	[1/μm^2^]	2.290 ± 0.479	2.620 ± 0.778	1.990 ± 0.401	3.700 ± 1.261	2.940 ± 0.254
Spc	[1/μm]	32561 ± 7825	30270 ± 9318	18006 ± 1691	36391 ± 11325	18643 ± 1490
S10z	[μm]	0.700 ± 0.088	0.705 ± 0.175	0.381 ± 0.050	0.413 ± 0.050	0.579 ± 0.094
S5p	[μm]	0.350 ± 0.041	0.319 ± 0.068	0.190 ± 0.030	0.225 ± 0.030	0.265 ± 0.045
S5v	[μm]	0.350 ± 0.050	0.386 ± 0.107	0.191 ± 0.021	0.188 ± 0.023	0.315 ± 0.054
Sda *	[μm^2^]	0.727 ± 0.225	0.508 ± 0.095	0.769 ± 0.114	0.721 ± 0.313	0.516 ± 0.033
Sha	[μm^2^]	0.471 ± 0.083	0.410 ± 0.093	0.575 ± 0.141	0.317 ± 0.112	0.365 ± 0.027
Sdv *	[μm^3^]	2.532 ± 0.535	2.709 ± 0.302	2.077 ± 0.684	3.293 ± 1.816	2.387 ± 1.065
Shv *	[μm^3^]	4.404 ± 1.249	4.063 ± 1.827	4.482 ± 2.068	1.565 ± 0.758	4.044 ± 1.313

* Samples without significant difference, one-way ANOVA and Tukey’s test (*p* < 0.05).

**Table 4:** Functional parameters of the thin films, according to ISO 25178-2:2012.

Par	Unit	Electrodeposition Time				
		2 s	4 s	6 s	8 s	10 s
***Functional***						
Smr *	[%]	0.002 ± 0.001	0.002 ± 0.001	0.002 ± 0.001	0.003 ± 0.002	0.003 ± 0
Smc	[μm]	0.155 ± 0.027	0.134 ± 0.027	0.087 ± 0.013	0.086 ± 0.006	0.111 ± 0.002
Sxp	[μm]	0.239 ± 0.032	0.237 ± 0.063	0.133 ± 0.019	0.132 ± 0.016	0.189 ± 0.016
Sk	[μm]	0.289 ± 0.054	0.231 ± 0.027	0.175 ± 0.024	0.166 ± 0.012	0.213 ± 0.011
Spk	[μm]	0.131 ± 0.019	0.121 ± 0.036	0.063 ± 0.013	0.076 ± 0.008	0.096 ± 0.038
Svk	[μm]	0.127 ± 0.018	0.147 ± 0.052	0.063 ± 0.008	0.070 ± 0.008	0.107 ± 0.016
Smr1 *	[%]	11.837 ± 2.197	12.263 ± 1.255	9.786 ± 1.317	10.899 ± 0.298	9.952 ± 0.361
Smr2 *	[%]	89.087 ± 2.088	87.854 ± 1.082	89.750 ± 0.913	89.708 ± 0.864	87.83 ± 0.929
***Volume***						
Vmp	[µm^3^/µm^2^]	6.265 ± 0.823	5.618 ± 1.510	3.205 ± 0.616	3.761 ± 0.462	4.798 ± 1.898
Vmc	[µm^3^/µm^2^]	105.80 ± 19.58	87.56 ± 13.97	62.73 ± 10.60	59.32 ± 5.25	78.29 ± 4.29
Vvc	[µm^3^/µm^2^]	147.50 ± 25.924	125.22 ± 24.05	82.89 ± 12.28	81.86 ± 5.82	102.37 ± 6.58
Vvv	[µm^3^/µm^2^]	14.241 ± 2.300	14.84 ± 4.327	7.504 ± 0.972	7.847 ± 0.837	11.49 ± 1.027

* Samples without significant difference, one-way ANOVA and Tukey’s test (*p* < 0.05).

**Table 5:** Hurst coefficient (H) of the TiO_2_ thin films with different electrodeposition times: 2, 4, 6, 8, and 10 s.

Par	Electrodeposition Time				
	2 s	4 s	6 s	8 s	10 s
H *	0.53 ± 0.04	0.51 ± 0.08	0.48 ± 0.07	0.41 ± 0.16	0.59 ± 0.04

* Samples without significant difference, one-way ANOVA and Tukey’s test (*p* < 0.05).

## Data Availability

The processed data required to reproduce these findings are available from the corresponding authors.

## Supplementary Materials

The following are available online at https://www.mdpi.com/article/10.3390/mi13081361/s1, Table S1: Calculated Rietveld refinement results for XRD measurements performed on electrodeposited TiO_2_ thin films.
