# Supplementary material for: Effect of the Deposition Time on the Structural, 3D Vertical Growth, and Electrical Conductivity Properties of Electrodeposited Anatase–Rutile Nanostructured Thin Films
Source: Micromachines (Basel). 2022 Aug 21;13(8):1361. doi: 10.3390/mi13081361 (PMC9412731; doi:10.3390/mi13081361)
Supplement: Supplementary file 1 [file micromachines-13-01361-s001.zip › micromachines-1858084-supplementary.pdf]

micromachines

MDPI

Article

Structural, vertical growth, and electrical conductivity properties of time-dependent electrodeposited Anatase-Rutile nanostructured thin films

Structural, 3D morphological, and electrical conductivity properties of electrodeposited TiO2 thin films.

Table S1. Calculated Rietveld refinement results for XRD measurements performed on electrodeposited TiO2 thin films.

	**Electrodeposition time**				
**Parameters**	2s	4s	6s	8s	10s
Crystal system	Tetragonal	Tetragonal	Tetragonal	Tetragonal	Tetragonal
Space group	I 41/a m d	I 41/a m d	I 41/a m d	I 41/a m d	I 41/a m d
a (Å)	3.79	3.77	3.71	3.79	3.79
c (Å)	9.52	9.45	9.54	9.53	9.53
Phase (%)	78.20	75.70	76.20	79.8	82.10
Crystallite size Anatase (nm)	13.07	12.97	12.55	14.39	15.09
Crystal system	Tetragonal	Tetragonal	Tetragonal	Tetragonal	Tetragonal
Space group	P 42/m a m	P 42/m a m	P 42/m a m	P 42/m a m	P 42/m a m
a (Å)	4.59	4,566	4.59	4,594	4,593
c (Å)	2.96	3,052	2.96	2,962	2,961
Phase (%)	8.50	7.40	16.00	13,0	11,9
Crystallite size Rutile (nm)	11,32	12,82	12,44	12,08	10,19
Crystal system	Cubic	Cubic	Cubic	Cubic	Cubic
Space group	I a -3	I a -3	I a -3	I a -3	I a -3
a (Å)	10.22	10,19	10.22	10.22	10.22
Phase (%)	13.30	17.00	7.80	6.20	6.10
R_{exp}	7.50	8.30	9.60	8.70	7.20
R_{wp}	12.30	11.7	13.50	15.20	10.30
